# Intervention effect of group counseling on social support and post-stress growth of orphans and vulnerable children in China

**DOI:** 10.3389/fpsyg.2022.962654

**Published:** 2022-08-25

**Authors:** Lyuci Zhang, Sumei Wu, Samsilah Roslan, Zeinab Zaremohzzabieh, Ye Chen, Yuqin Jiang

**Affiliations:** ^1^Department of Education and Music, Hezhou University, Hezhou, China; ^2^Department of Education, Guangxi Normal University, Guilin, China; ^3^Department of Foundation Studies, Faculty of Educational Studies, Universiti Putra Malaysia, Serdang, Malaysia; ^4^Faculty of Human Ecology, Universiti Putra Malaysia, Serdang, Malaysia; ^5^Faculty of Human Development, Universiti Pendidikan Sultan Idris, Tanjung Malim, Malaysia

**Keywords:** social support group, psychological counseling intervention, post-stress growth, social support, orphans

## Abstract

Orphans and vulnerable children fall under the category of children who are at risk of exposure to more stressful circumstances and receive less social assistance compared to other children. This study aims to investigate the impact of group counseling based on social intervention and psychological therapy (SSGPC) on social support and the perceived stress growth of orphans and vulnerable children. In one special educational needs school in Nanning, China, the SSGPC was developed and implemented. Using the social support and post-stress growth scales, the researchers investigated the effects of SSGPC on orphans and vulnerable children. Twenty-seven orphans and vulnerable children between the ages of nine and 12 were arbitrarily assigned to the experimental and control groups. A pre-test post-test method of quasi-experimental design was applied, with 13 participants in the experimental group and 14 in the control group. The results revealed that the intervention group had significantly higher scores for social support and post-stress growth than the control group. The SSGPC had significantly improved the levels of social support for orphans. The findings indicated that the SSGPC provided an effective way to improve social support and post-stress growth of orphans and vulnerable children.

## Introduction

China is now witnessing fast economic expansion. According to Liu et al. (2020), this expansion aided the Chinese society in improving its living standards in many areas, including a reduction in the number of impoverished people, a lower rate of unemployment, and a reduction in the number of children without a family. According to Shang and Cheng (2006), the total number of orphans in China was 573,000. In 2020, China’s orphan population was expected to be at 193,300, down from over 233,000 the previous year (National Bureau of Statistics of China, 2020). Accordingly, orphans and vulnerable children (OVC) are more likely to suffer from hunger, drop out of school, suffer from low psychological wellbeing, and have an earlier sexual debut (Yendork, 2020). Specifically, among OVC, poor mental health functioning has been extensively proven to be connected with various detrimental health and social repercussions, even into adulthood (Scott et al., 2016). According to Chi et al. (2014), OVC may not communicate their anxieties and worries, resulting in sentiments of wrath, resentment, as well as a sense of estrangement, and despair. Such emotions can result in risk-taking behaviors and withdrawal. Furthermore, severe negative emotions can lead to aggression or various problematic behaviors (Loney et al., 2006).

Scholars have previously demonstrated that OVC are vulnerable to deprivation as a result of mistreatment and isolation from siblings, putting them at risk for mental health issues (e.g., Morantz et al., 2013). It is concerning that OVC’s psychological wellbeing receives little attention, since previous academics have recognized that few evidence-based initiatives have addressed these children’s mental health and behavioral health preventative requirements (Mellins and Malee, 2013; Skeen et al., 2017). Caspe and Lopez (2006) showed that interventions that promote family involvement in children’s development build parental knowledge and skills, and improve family stability through, for example, efforts toward economic security and social inclusion that fall under the rubric of “family strengthening.” Nevertheless, it is critical to stress that social support (SSU) should be offered to OVC as early as possible when a lack of parent and family instability begin to have some of the most detrimental consequences on their life (Doku et al., 2019). SSU appears to have acquired some momentum, according to existing studies on the wellbeing of children placed in orphanages (Zhou, 2012).

In addition, post-stress growth (PSG) is an important variable for OVC. After undergoing stressful or traumatic circumstances, PSG is described as recovering psychological equilibrium and further growing psychological maturity (Hu et al., 2021). Losing a parent and being placed in an orphanage can be traumatic because it changes one’s status in life (Zhang et al., 2022). There are many ongoing stressful and traumatic life events for OVC such as abuse, neglect, and parental loss among others (Nyathi, 2022). Additionally, vulnerable children and their families commonly live in a state of poverty (Marais et al., 2014). As a result, vulnerable children experience more stressful events than the average children do. However, from the perspective of positive psychology, positive changes within individuals are explored to produce more positive results. Therefore, this study examines the pressure on OVC from a positive perspective. When OVC experiences pressure or negative events, positive changes are explored to achieve growth after pressure.

Fan and He (2016) discovered that group counseling can work as an intervention to prevent the development of severe mental illness. Group counseling has consistently been shown to increase SSU and PSG of OVC (Fawzi et al., 2012; Sitienei and Pillay, 2019; Penner et al., 2020). Group counseling is a tool in which several OVC participate in social intervention and psychological therapy (SSGPC) to help them change or deal with a long-term problem they are experiencing, guided by a therapist or counselor (Gidron, 2020). The SSGPC has many benefits and is thus appropriate for a variety of difficulties, particularly those OVC with interpersonal concerns. Stress management, for example, is one of them. The SSGPC, which is based on Bronfenbrenner’s (1979) ecological systems theory (EST), is designed to help OVC in a culturally appropriate and effective manner. According to EST, human development is mostly influenced by how people interact with their environment as it changes (Senefeld and Perrin, 2014). All ecological systems have a significant impact on OVC’s growth, favorably either if support systems are present or adversely if they are not. If the microsystem, particularly the family, fails, the OVC may be unable to explore other ecological systems unless they acquire group counseling. The SSGPC may be viewed as an alternative ecological system that can support the OVC in the shortest amount of time. The SSGPC is likely to provide the OVC with psychological and SSU services that their families, schools, or even communities are unable to provide. This indicates that the SSGPC must take into account a comprehensive strategy that can raise OVC’s SSU and PSG. By situating the study’s proposed work inside the ecological systems theory, the OVC’s PSG and SSU may benefit to some extent from the known SSGPC.

Nevertheless, it should be highlighted that many OVC programs prioritize meeting children’s basic needs over their non-material needs, such as providing mental support and stress relief (Onuoha and Munakata, 2010). Community development may be used to treat OVCs’ mental health, but in many circumstances (Cheney, 2010), OVCs in South Africa may not have enough access to this type of mental health care. (Marais et al., 2014). In light of this, the current study seeks to determine if the SSGPC is effective in improving the SSU and PSG of OVC in the context of China.

## Materials and methods

### Participants and procedure

In this study, participants were recruited by voluntary enrollment in a school for OVC, and selected according to scale tests and interviews. The criteria for selecting the intervention group were individuals who: (1) could attend each group counseling activity on time; (2) were in good health and had no difficulties in language expression and communication; (3) had low scores on the SSU scale; and (4) had the motivation to change and willingness to participate in group counseling. After the interview, 27 eligible subjects were randomly assigned to the intervention group and the rest were set as members of the control group, namely 13 in the intervention group and 14 in the control group. All the children went to boarding school together, and the two groups of subjects possessed the same family situation and study life. The age range was 9–12 years old, and the grades were from Grade 3 to Grade 6. Before beginning the interventions, caregivers and parents provided written consent for their own participants.

The intervention was held from October 12, 2021 to December 28, 2021. A group counseling session was held every Saturday from 3.30 pm to 5.30 pm for a total of six times. The venue was the psychological counseling room of the OVC’s school. The participants in the experimental group underwent the SSGPC program, while the participants in the control group engaged in activities, including games, coloring activities, and singing songs. During the intervention period, 13 members of the intervention group participated in six of the SSGPC activities completely and filled in the summary of each intervention activity, experience, and group counseling effect evaluation form. After the group counseling session, each participant completed the SSU and PSG scales for the post-test.

### Instruments

#### Social support scale

The youth SSU scale prepared by Ye and Dai (2006) was adopted. The scale contains 17 items and three dimensions: subjective support, objective support, and support utilization. The five-point Likert scale was used. It has been shown to be reliable (Cronbach’s α = 0.82; Zhang et al., 2022) and valid for measuring the social support of the Chinese population (Liu et al., 2011).

#### Post-stress growth scale

Qin and Wu’s (2016) scale was employed to develop the children’s PSG scale. The scale is self-rated and measures positive changes in the past year. There are 15 items on the scale, with five items in each dimension, including coping style, interpersonal relationship, and life philosophy. The Likert self-rating scale was scored with 5 points, ranging from 1 for “very inconsistent” to 5 for “very consistent.” The item average score was used as an indicator of individual growth level after stress. The higher the score, the higher the individual growth level after stress. The reliability and validity of PSG were 0.737 and 0.727, respectively (Qin and Wu, 2016).

#### Social support group psychological counseling intervention program

Based on a questionnaire survey, current situation analysis, and literature search, and following the principle of group dynamics with regard to the formulation of a group counseling program (Fan and He, 2016), six units of group counseling activities were designed in this study as outlined in Table 1. In this study, group dynamics could sometimes interfere with the OVC group’s ability or willingness to share their true thoughts, which they did more of in individual therapy. Each child brought to the group a particular personality, life experiences, fears, perceptions, gender influences, ethnicity, prejudices, and cultural individualities. Therefore, standard errors might have been underestimated (Paddock et al., 2011). It would be impossible to discuss all of the differences or influences that defined the children in the group sessions, but there were a few key group differences that seemed to underscore how a person interacted with others in a group (Deci and Ryan, 2000; Luyckx et al., 2009). In this study, strategies to reduce all of the differences included explaining the purpose and role of group therapy to the children before the group session started, adjusting and conducting thorough screening of the OVC group, asking the children to create group rules that would make the group safe and productive for the OVC group, and offering individual therapy in conjunction with the group for those who required it. Clinical services offering group interventions could consider providing training on managing group dynamics to staff facilitating groups in order to manage this important component of group interventions.

**Table 1:** Intervention activities.

**Goal**	**Activities**	**Goal**	**Activities**
**Unit1 1.** 1. Set up a team and make a team pact collectively 2. Team members get to know each other, get familiar with each other, establish interactive relations, and feel the warmth of the team	1. Warm-up activity: smile and shake hands. Mentor: “Today you are different from the previous you. You are happy and optimistic today, so that everyone in our group can feel the different you today. A. All members sit in a circle B. Give everyone a minute to introduce himself or herself. C. Sharing: teachers guide members to think and discuss. 2. Circle sit, choose a hand holding newspaper roll into the “stick,” the director shouted a member of the nickname, called the left and right sides of the members to immediately stand up, otherwise by called to give a blow, “stick thin lover,” repeatedly do, until everyone familiar with each other’s name. 3. Who can remember the most names of other people.	**Unit 2.** 1. Promote self-cognition and strengthen self-understanding 2. Increase the understanding of others, better understand others, affirm others, and promote interpersonal interaction	1. Take out the mirror and take a closer look at yourself, as students may not have this opportunity to take a closer look at themselves. Then the teacher asked the students to think for a few minutes and say what kind of person they are. 2. Each person writes 20 sentences “I am a XXXXXX person.” Ask to reflect the characteristics of the individual, after writing fixed group communication, everyone holds the mood of understanding others, to get to know each unique person in the group. Finally, the group representative spoke and the group shared their feelings. 3. The teacher first presented the activity’s rules: divide the students into groups of 3–5, give them each a small piece of paper, and have them write down their thoughts on the other students in the group. Students are not permitted to speak during the activity. Instead, they can only jot down on paper what they wish to say to the interviewee. They can share their thoughts and provide ideas.
**Unit 3**. 1. Let members learn the mode of effective communication between classmates 2. To encourage students to learn empathy	1. The rules of the game are as follows: Each team member must jump off a 1.6-meter platform straight back while his teammates hold out their hands to shield him. To avoid a lack of security, everyone wants to be able to trust one another. Be trustworthy if you want people to trust you. Getting people to trust you might be challenging when they have suspicions about you. Through the course of the game, teammates can enhance their sense of responsibility and trust for one another. 2. Teachers lead the team members on a tour of the school and provide them instructions to get there without any problems. The team can test their communication, cooperation, tacit understanding, empathy, and teamwork while walking. They can also test their listening and communication abilities when they run into obstacles or move too quickly.	**Unit 4.** 1. Learn to understand and respect teachers 2. let members through personal experience to perspective-taking Improve members’ understanding and comprehension of teacher support	1. let all the members to hand circle at the beginning, then, leaders said that “, a group of four “, members must, in accordance with the requirements of the gang of four again, to form a new “home,” at the moment, please find home to talk to the person who do not have the feeling of the free outside groups, mostly talks to “lonely, lonely, abandoned, do not rely on, lost, worried about…” You can also ask members of the group to share their feelings of being with you. Most of them will say “warm, powerful, safe, dependable…” The number of members can be changed many times, so that members have the opportunity to change their behavior, actively integrate into the group, so that members can experience the feeling of home, experience the support of the group, so that they are more willing to stay with the group. 2. Prelude: Play the song “Get You” or “It’s Morning” Prepare 3 small scenes, pen and paper and 2–3 students discuss the topic situation, make and act out a corresponding situation, other students think in others’ shoes, understand others, For example, the situation: always gentle mother came back from work today, sad face, temper is very angry, because of a little thing on xiaoming angry. Xiaoming is very distressed very aggrieved, hence… Four steps of perspective-taking: Step 1: If I were him, WHAT I’d need is… Step 2: If I were him, I would not want to… Step 3: If I were the other person, what I would do is… Step 4: Am I treating him the way he expects me to? 3. Sharing Summarize and fill in the group experience form.
**Unit 5.** 1. Experience and understand their loved ones pay and care for themselves 2. Strengthen the ability of empathy 3. improve the ability to understand teacher support	1. Each group circle, invite one member to the middle, other members hand in hand circle. At the beginning of the exercise, the circle members close their eyes, consciously and comfortably lead to any side, other members must join hands to form a protective circle for protection, cannot let the circle members fall down. Where he falls, the group goes to catch him, protect him, and push him to the center. Therefore, fall, catch, the middle member from nervous to very relaxed. Can switch to the circle to experience. The activity fully embodies the cooperation of the group. 2. Sharing: What went through your mind when you fell down? People outside the circle How do you feel when you pick someone up? (Thank you for your trust and help.) 3. Write the stories of teachers who have influenced me greatly and share them with each other. Be as specific as possible. 4. share 5. Read the material aloud and let the members close their eyes, hold hands with each other and form a circle. The leader will read the words of gratitude (materials), and let the members engage in meditation and memory as much as possible. 6. Express gratitude to a teacher or classmate by text message or email, or by phone	**Unit 6.** 1. Consolidate learning experience 2. End group counseling, return to reality, look into the future, and encourage members to apply what they have learned to real life.	1. To bring warmth and strength through physical contact, so that members can confirm the unity of the group more realistically before the end, experience the feeling of being together, and gain support and confidence. 2. How to do it: at the end of the last group activity, the instructor asks everyone to stand in a circle, put your hands on the shoulders of each group member, and gather together in silence for 30 s. Then gently hum the familiar song, and sway freely with the melody of the song. From children’s songs to country songs, try to find people who know them, all of them, one after another. Let all members bid farewell to the group in a warm, sweet and cohesive scene, and walk towards life, leaving a forever, beautiful, very symbolic, unforgettable memory. 3. Parting 4. Give gifts 5. Fill in the questionnaire 6. Take a group photo

## Data analysis

Before the first group counseling, SSU and PSG scales measured all members of the intervention group and control group. All data were analyzed with IBM SPSS Statistics 22. The normal distribution test showed that the data in this study conformed to normal distribution, while the parametric test was applicable. Therefore, independent samples t-test and paired samples t-test were run to assess between-groups differences and within-groups differences in the SSU and PSG scales before and after SSGPC, respectively. Additionally, effect sizes *d* were calculated (small ≥0.20, medium ≥0.50, and large ≥0.80; Cohen, 1988).

## Results

### Between-subjects analyses

**Figure 1:**
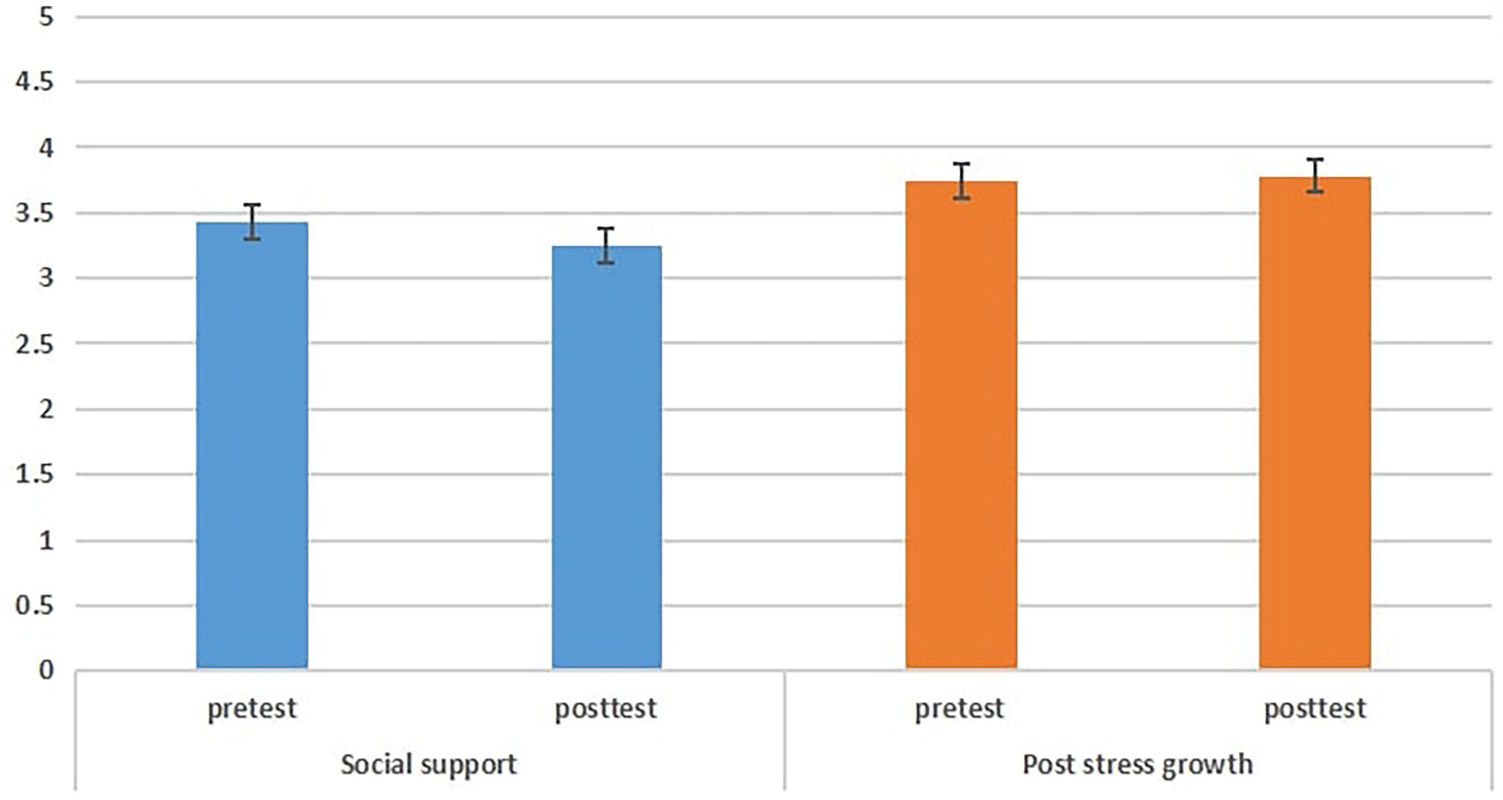
Comparison of pretest and posttest differences in the control group.

The independent samples t-test revealed no differences for SSU before SSGPC (see Figure 1); however, a large effect of between-groups difference in SSU [*t* = −10.181, *p* = 0.000, *d* = 3.98] and PSG scores [*t* = −2.791, *p* = 0.000, *d* = 3.59] was found. Therefore, pre-test and post-test differed in improving SSU and PSG after SSGPC with higher SSU and PSG scores in the experimental group than in the control group (see Table 2).

**Table 2:** Mean and standard deviation of social support and post-stress growth before and after intervention.

	Experimental group (*N* = 13)	Control group (*N* = 14)	Independent samples *t*-test	Paired samples *t*-test		Experimental group (*N* = 13)	Control group (*N* = 14)	Independent samples *t*-test	Paired samples *t*-test		Experimental group (*N* = 13)	Control group (*N* = 14)
	Before intervention	After intervention	Before intervention	After intervention		Before intervention	After intervention	Before intervention	After intervention		Before intervention	After intervention
	***M(SD)***	***M(SD)***	***M(SD)***	***M(SD)***		***M(SD)***	***M(SD)***	***M(SD)***	***M(SD)***		***M(SD)***	***M(SD)***
SS	2.99(0.24)	4.05(0.35)	2.95(0.20)	2.89(0.23)	SS	2.99(0.24)	4.05(0.35)	2.95(0.20)	2.89(0.23)	SS	2.99(0.24)	4.05(0.35)
PSG	3.19(0.20)	4.30(0.36)	3.08(0.23)	3.18(0.19)	PSG	3.19(0.20)	4.30(0.36)	3.08(0.23)	3.18(0.19)	PSG	3.19(0.20)	4.30(0.36)

SS, Social support; PSG, Post-stress growth

### Within-subjects-analyses

No differences in SSU and PSG were found. Nevertheless, the paired samples t-test (see Table 2) revealed a large effect increase of SSU [*t* = −11.599, *p* = 0.00, *d* = 3.221] and PSG [*t* = −11.921, *p* = 0.001, *d* = 3.259] scores in the experimental group but not in the control group. Therefore, the results indicated that the SSGPC should take into account a group counseling program that could increase OVCs’ SSU and PSG.

### Evaluation of group intervention effect

The feedback sheet of the group activity effect was used in this study to design five topics. The results indicated that the contents of the items in the table matched the theme and content of group psychological counseling activities. The scoring scale was 0 to 10, with 0 representing extremely dissatisfied, and 10 representing extremely satisfied. The higher the score, the more satisfied. It was employed to examine group members’ overall experience and evaluation of counseling activities. The subjective evaluation of intervention group members on group activities is shown in Table 3.

**Table 3:** Group psychological counseling effect feedback sheet.

**No.**	**Item**	**1st** **(M)**	**2nd** **(M)**	**3rd** **(M)**	**4th** **(M)**	**5th** **(M)**	**6th** **(M)**
1	I appreciate the corporate atmosphere of this group	8.92	9	9.25	9.92	9.61	9.53
2	I like the content of this group activity	8.46	8.92	9.61	9.84	8.92	9.84
3	I agree with the leadership of this group activity	8.53	9.84	9.53	9.3	9.23	9.76
4	I agree with the leadership of this group activity	8.92	9.46	8.92	9.53	9.84	9.61
5	I took an active part in this group coaching activity	8.00	9.38	9.61	9.15	9.61	9.84

## Discussion

The current study examined the intervention effects of SSGPC on SSU and PSG with a focus on OVC in the context of China. The results of this study provided proof that the SSGPC offered psychoeducational and social interventions to OVC. According to the study, group counseling was used to support the SSU and PSG of OVC to help them cope with the psychological difficulties they were experiencing. One of the key strengths of the SSGPC is that it is based on Bronfenbrenner’s (1979) ecological model. Each child is viewed by the SSGPC as a dynamic individual who is both impacted by and able to influence many systems. Masten (2001) argued that one must concentrate on both the child and the system in which the child lives if one wants to minimize stress or increase resilience through intervention. The SSGPC considers these processes and seeks to improve them, intervening in a more comprehensive and efficient manner. To the authors’ knowledge, this is the first study to look at SSGPC’s efficacy with the OVC population in China.

The first finding of this study showed that there was a significant increase in SSU in the intervention group as compared to the control group. The findings of this study regarding social and peer support concurred largely with those documented by earlier researchers (Kumakech et al., 2009; Hong et al., 2010; Doku et al., 2015; Li et al., 2019). Within the SSGPC, the OVC discovered that they did not live in isolation, but belonged to a social system. These outcomes concurred with those from a study in Rwanda by Lavin et al. (2010), who found that intervention involving group counseling improved children’s social support. Based on the ecological systems theory, Mc Guckin and Minton (2014) maintained that the environment in which a child grows up plays a critical role in shaping the relationship between the child and their development. As such, the SSGPC is helpful for OVC in enhancing mental health and social interaction, and establishing new relationships with their community, family, and peers.

Additionally, the results revealed that there were significant differences in the levels of PSG between the pre-test and post-test in the intervention group, indicating that the PSG in the intervention group was significantly higher than those before the intervention. Therefore, the SSGPC is effective in improving the level of PSG of OVC. The results of this study were in line with those of an earlier study, which showed that trauma-focused cognitive behavior therapy and cognitive behavioral interventions for trauma in schools could enhance PSG in children who had experienced trauma (Little et al., 2011). Regarding findings, teachers gave their positive feedback, which illustrated that the SSGPC might be effective in post-traumatic stress disorder (PTSD) symptom reduction. Meanwhile, the SSGPC’s supportive mechanisms and effective teamwork were thought to be crucial for raising the level of PSG of OVC. Through the six SSGPC activities, it can be seen that the group psychological counseling to each member of the help was very significant. This study can provide some reference value for OVC’s mental health and intervention.

Regardless of the context, the SSGPC reawakens awareness of OVC, highlighting their needs and mobilizing assistance for them. An important factor of the SSGPC is that it was run in a community-based school, thus promoting awareness of OVC to the teachers and principal in the school and offering the staff support and contact with other professionals who are interested in and trained to assist OVC. The findings of this study regarding training of teachers in schools to acquire the basic skills required to support OVC concurred with earlier studies on the same issue (Wood and Goba, 2011). Therefore, training all teachers in terms of SSGPC would enable them to cope with the large numbers of OVC that are found in schools.

## Limitations and future research directions

This study has a few limitations that should be addressed. First, because this was an interventional study, it was unable to draw firm conclusions from the connection between variables. As a result, follow-up and longitudinal studies may be conducted in the future to address this issue. Second, because all of the primary factors were self-reported, self-presentation biases may have altered the connections between the variables. Future research might benefit from obtaining data from numerous respondents to overcome these possible biases (e.g., teachers, orphanage staff). Third, the focus of this study was primarily on the effect of SSU group psychological therapy intervention on SSU and PSG. More research is needed to investigate other parameters that influence the SSU and PSG of Chinese OVC.

## Conclusion

In this study, the SSGPC was adopted to improve the level of SSU and PSG of OVC. For OVC, increased levels of SSU are necessary to improve their mental health. The results showed that this group counseling intervention effectively improved the level of SSU for OVC. In addition, this study further explored the potential positive effects of the effect of SSGPC on stress after growth. It was found that the intervention improved the PSG level by improving the level of SSU. The results of this study provided some insight into the prevention and intervention of OVC’s mental health. Specifically, the content of the intervention and the form of intervention can be designed to prevent OVC from the negative effects of low SSU. It can also be that OVC are more likely to grow up after experiencing stressful events. Therefore, this intervention provides a certain direction for improving the SSU and PSG of OVC.

## Data availability statement

The original contributions presented in the study are included in the article/supplementary material, further inquiries can be directed to the corresponding author.

## Author contributions

LZ and SR: conceptualization and writing—original draft preparation. LZ and YJ: methodology. LZ and YC: software. SR: validation and supervision. LZ: formal analysis and visualization. YJ: investigation. SW and SR: resources. LZ and ZZ: data curation. ZZ: writing—review and editing. All authors contributed to the article and approved the submitted version.

## Conflict of interest

The authors declare that the research was conducted in the absence of any commercial or financial relationships that could be construed as a potential conflict of interest.

## Publisher’s note

All claims expressed in this article are solely those of the authors and do not necessarily represent those of their affiliated organizations, or those of the publisher, the editors and the reviewers. Any product that may be evaluated in this article, or claim that may be made by its manufacturer, is not guaranteed or endorsed by the publisher.
